# Effect of *APOE* and *CD33* on Cognitive Decline

**DOI:** 10.1371/journal.pone.0130419

**Published:** 2015-06-23

**Authors:** Kathleen M. Hayden, Michael W. Lutz, Maragatha Kuchibhatla, Cassandra Germain, Brenda L. Plassman

**Affiliations:** 1 Department of Psychiatry and Behavioral Sciences, Duke University Medical Center, Durham, North Carolina, United States of America; 2 Joseph and Kathleen Bryan Alzheimer’s Disease Research Center, Duke University Medical Center, Durham, North Carolina, United States of America; 3 Department of Neurology, Duke University Medical Center, Durham, North Carolina, United States of America; 4 Department of Biostatistics, Duke University Medical Center, Durham, North Carolina, United States of America; Taipei Veterans General Hospital, TAIWAN

## Abstract

**Objective:**

An Alzheimer’s disease (AD) diagnosis is preceded by a long period of cognitive decline. We previously demonstrated increased risk of decline among individuals possessing one or more *APOE* ε4 alleles together with a family history of AD. The objective of this study is to investigate the possibility that such an increased risk might be due to AD risk genes with small effects in combination with *APOE*.

**Methods:**

Participants in the Health and Retirement Study (HRS) over the age of 65, who contributed DNA, and had two or more evaluations with an abbreviated version of the modified Telephone Interview for Cognitive Status (TICS-m) were eligible for the study (n = 7451). A genetic risk score (g-score) was derived using AD risk genes’ meta-analyses data, assigning risk according to the number of risk alleles and summed over all the risk genes. Trajectories of cognitive function were modeled in four groups of Caucasian participants with and without one or more *APOE* ε4 alleles and either a high or low g-score: *APOE* ε4-/low g-score; *APOE* ε4-/high g-score; *APOE* ε4+/low g-score; and *APOE* ε4+/high g-score. Post hoc analyses evaluated interactions between individual genes and *APOE*.

**Results:**

Individuals in the *APOE* ε4+/high g-score group exhibited the greatest cognitive decline over time (p<.0001). This risk appeared to be greater than the sum of the effects of either high g-score or *APOE* ε4 alone. When gene interactions were individually tested with *APOE*, a statistically significant interaction with *CD33* was discovered (p = 0.04) although the interaction was no longer significant when adjusted for multiple comparisons.

**Conclusions:**

Individuals with multiple AD risk genes in addition to having one or more *APOE* ε4 alleles are at greater risk of cognitive decline than individuals with either *APOE* ε4 or a high genetic risk score. Among those with one or more *APOE* ε4 alleles, having one or more copies of the *CD33* C (risk) allele may further increase the risk of cognitive decline.

## Introduction

An Alzheimer’s disease (AD) diagnosis is preceded by a long period of cognitive decline. During this time when cognitive changes are relatively mild, it may be possible to alter the trajectory of cognition. Researchers are seeking to identify and recruit into clinical trials, individuals who may be on a downward trajectory in order to test new therapeutic treatments and interventions. In some cases, those at highest genetic risk are recruited (*e*.*g*., individuals with *PSEN1*, *PSEN2*, or *APP* mutations), however this approach samples from a narrowly defined and relatively small group of individuals who are at the highest risk of early onset Alzheimer’s disease (EOAD). A number of genes with small effects have been identified as risk factors for late onset AD in a number of large, multi-cohort GWAS studies [1–4], so it stands to reason that individuals possessing multiple risk alleles have an even greater risk of AD and may be more likely to express an endophenotype of cognitive decline than those possessing fewer risk alleles. To date, little work has been done to demonstrate this potential association.

We previously reported the effects of family history of dementia and the *APOE* ε4 allele on cognitive performance over time with data from the Cache County Study of Memory Health and Aging (CCSMHA)[5]. In that study, family history was presumed to represent some combination of genetic predisposition or shared environment. Individuals possessing one or more *APOE* ε4 alleles declined faster than those without *APOE* ε4, and individuals who had a family history of dementia in addition to one or more *APOE* ε4 alleles had the worst performance over time on average. We now seek to test the hypothesis that other AD risk genes contribute to cognitive decline in a manner similar to the CCSMHA Study but now using genes instead of an endorsement of family history of AD. Specifically, we hypothesize that the combination of *APOE* ε4 and AD risk genes will be associated with a steeper downward trajectory of cognitive function.

## Methods

Data for the study were drawn from the Health and Retirement Study (HRS) [6]. The HRS began data collection in 1992 and the Asset and Health Dynamics among the Oldest Old (AHEAD) study began in 1993. The two samples were merged in 1998 and are now referred to as HRS. Additional cohorts were added, including the War Baby study and the Children of the Depression study. Together, these studies provide comprehensive biennial data on a large sample that is representative of the U.S. population aged 50+. Interviews are conducted with all HRS respondents every two years by the Survey Research Center at the University of Michigan. All participants provided informed consent. Consent is inferred by the completion of mailed surveys; oral consent is obtained for telephone interviews; and written consent is obtained for biological samples. The study protocol, methods, and consent procedures were approved by the University of Michigan Institutional Review Board (IRB); the current secondary data analysis was approved by the Duke University Medical Center IRB. Interviews are conducted primarily by telephone, except for in-person interviews to cover specific sub-studies and with those aged 80 and older.

### Participants

Participants for the current study are members of the HRS aged 65+, who provided genetic material for GWAS and completed HRS Telephone Interview for Cognitive Status-modified (TICS-m). Our sample is limited to participants aged 65+ because beginning in 1998, the HRS TICS-m was administered only to those age 65+ and participants not interviewed at the prior wave. If an individual was initially evaluated prior to age 65, only data from visits occurring after age 65 were used. To control for confounding due to population genetic substructure we focused on Whites only. Basic demographic information including sex and years of education were recorded as part of the baseline interview.

### DNA Samples and Selection of SNPs

Saliva samples were collected from HRS participants beginning in 2006. GWA analyses were completed in 2013. The Illumina HumanOmi1-Quad and Illumina Human Omni-2.5 Quad bead chips were used as genotyping platforms. Genetic data are available through dbGaP. In order to facilitate comparison with other platforms, imputation strategies were used to increase the number of available markers. SNPs that best represent the genes of interest were identified in the GWA data.

### Description of Variables

#### Genetic Risk Score

AD risk genes that were reported in a series of Nature Genetics papers from 2009–2011[1, 2]^,^[3, 7] and represent the “top 10” AD risk genes in the Alzgene resource were selected [8]. Genes included: *CLU*, *CR1*, *PICALM*, *MS4A6A/MS4A4E*, *CD33*, *CD2AP*, *ABCA7*, *CR1* and *PICALM*. *APOE* was considered separately.

A genetic risk score (g-score) was computed using the odds ratios for AD risk reported in published meta-analyses on the AlzGene website [8]. Odds ratios less than 1 were converted so that all the risks were reported in the same direction. The number of alleles possessed by each participant was multiplied by the odds ratio for each AD risk gene. The g-score is the sum of all the risks weighted by the strength of the reported odds ratio. The resulting continuous risk score was then dichotomized at the median into high and low scores. A separate variable was created based on quartiles of risk.

#### Genetic risk groups

Four mutually exclusive risk groups were determined based on *APOE* ε4 and g-score status. *APOE* was modeled separately because the strength of its association with cognitive function may mask other genetic effects. The reference group had no *APOE* ε4 and a low g-score (*APOE*-/low g-score), other groups included those with no *APOE* ε4 and a high g-score alleles(*APOE*-/high g-score), *APOE* ε+ and a low g-score(*APOE*+/low g-score), and the highest risk group with *APOE* ε4+ and a high g-score(*APOE*+/high g-score).

#### HRS TICS-m for identification of Cognitive Decline

The Telephone Interview for Cognitive Status (TICS) has been used extensively. The original version of the TICS [9], was modeled after the Mini-Mental State Examination (MMSE)[10] and has good discriminability between normal and demented elderly[11]. The HRS TICS-m, an abbreviated version of the modified version of the TICS, has a total of 35 points: 10 points each for immediate and delayed recall; 10 points for counting backwards, naming, and orientation; and 5 points for serial 7s. The HRS TICS-m has been administered serially since 1993 in the AHEAD sample and since 1996 in the HRS sample. The measure has been cross-validated for administration by telephone or in person[12]. Age-related changes in longitudinal performance on the HRS TICS-m have been reported[13] and are similar to other cognitive measures. If respondents were unable to complete the HRS TICS-m, a proxy informant was administered the IQCODE[14] to obtain an assessment of cognitive and functional decline of the respondent in the preceding two years. Only individuals with HRS TICS-m scores were included in the analysis.

### Analytic Approach

Demographic characteristics of the sample based on the four mutually exclusive genetic risk groups described above were compared with chi-square tests or generalized linear models (SAS PROC GLM). Repeated measures fixed effects models are used to estimate cognitive trajectories[15] using the SAS PROC MIXED procedure [16]. The dependent variable for the main analysis was a continuous measure of decline on the TICS-m. With this approach, individual differences in cognitive performance over time are captured while accounting for the correlation in repeated measures. Models were adjusted for baseline age, sex, and years of education. Age was used as the time scale and a quadratic term for age was tested to allow for non-linear progression of cognitive decline. Post hoc analyses evaluated interactions individually between each gene and *APOE* using similar mutually exclusive four-level factorial variable models and interaction terms.

## Results

**Fig 1:**
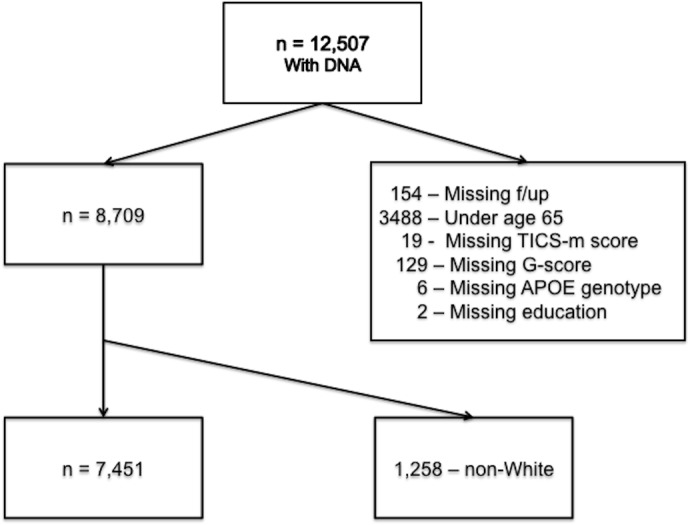
Sample Selection Flowchart. Abbreviations: f/up: follow-up; TICS-m: Telephone Interview for Cognitive Status-modified; G-score: genetic risk score.

A total of 8,709 individuals who contributed DNA and cognitive assessments after age 65 were identified from a total of 12,507 participants who provided DNA for genetic analysis (Fig 1). 7,451 of these self-identified as White and had complete data for this analysis. Demographic characteristics of the sample by genetic risk group are shown in Table 1.

**Table 1:** Demographic Characteristics of HRS Sample.

Characteristic	Group1 *APOE* ε4+ G-Score high	Group2 *APOE* ε4-G-Score high	Group3 *APOE* ε4+ G-Score low	Group4 *APOE* ε4-G-Score low	Total
**N (%)**	723 (9.7)	2783 (37.4)	840 (11.3)	3105 (41.7)	7451
**Mean # Observations(SD)** *	4.9 (2.4)	5.1 (2.4)	5.0 (2.4)	5.2 (2.4)	5.0 (2.4)
**Mean Baseline Age (SD)**	67.4 (3.3)	67.7 (3.6)	67.7 (3.8)	67.8 (3.7)	67.7 (3.7)
**Sex: Female (%)**	424 (58.6)	1561 (56.1)	475 (56.6)	1790 (57.7)	4250 (57.0)
** Male (%)**	299 (41.4)	1222 (43.9)	365 (43.5)	1315 (42.4)	3201 (43.0)
**Mean Yrs Education (SD)**	12.7 (3.2)	12.5 (3.1)	12.6 (3.1)	12.6 (3.0)	12.6 (3.1)
**Mean Baseline TICS-m (SD)**	23.7 (4.4)	23.8 (4.3)	23.7 (4.3)	24.0 (4.2)	23.8 (4.3)

Abbreviations: G-score: genetic risk score HRS: Health and Retirement Study; N: number; SD: standard deviation; TICS-m: Telephone Interview for Cognitive Status-modified; Yrs: years.

*There were statistically significant differences in the mean number of visits between the reference Group (Group 4) and Groups 3 and 1. There were also significant differences between Groups 1 and 2.

**Fig 2:**
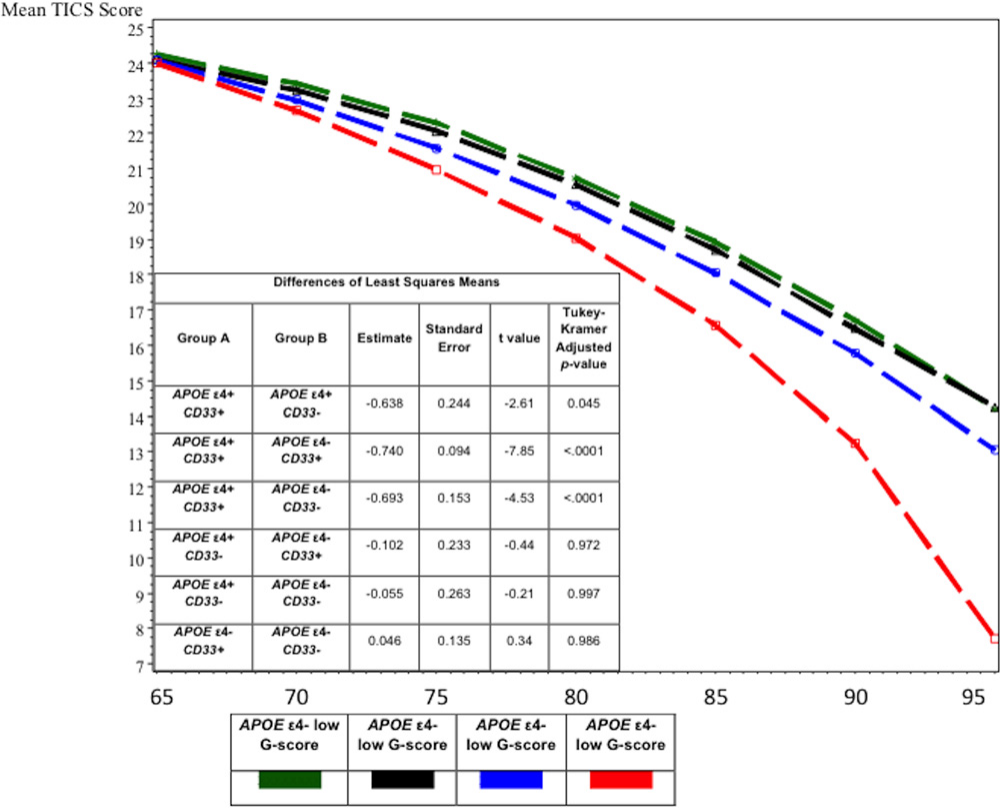
Mixed effects models for cognitive trajectories by *APOE* ε4/G-score risk group. Abbreviations: TICS-m: Telephone interview for Cognitive Status-modified; high G-score: high genetic risk score; low G-score: low genetic risk score. Model included age, age^2^, sex, sex*age, education, education*age, and group*age. Corresponding model results for the four-level variable and interaction terms are presented in Table 2.

A compound symmetry correlation structure best fit the repeated measures models. Models were constructed using the four risk groups described above and adjusted for baseline age, age*age, sex, sex*age, education in years, and education*age. The four-level factorial variable was also multiplied by age. The results for the four-level variable and age interaction are presented in Table 2. Individuals in the highest risk group (*APOE* ε4+/high g-score) had statistically significantly greater decline than individuals with neither risk factor (*APOE* ε4-/low g-score -0.11, 95% CI -0.13 to -0.08) (Table 2). The high-risk group also had greater decline than the sum of either genetic risk factor alone (Fig 2). Results were similar when we restricted the sample to just the highest and lowest quartiles of genetic risk score.

**Table 2:** Mixed Effects Models for Cognitive Decline by *APOE*/G-score group.

	Group		Group*Age	
	B(se)	*p-value*	B(se)	*p-value*
**APOE ε4+/G-Score high**	-0.075 (0.15)	0.6245	-0.106 (0.012)	<.0001
**APOE ε4+/G-Score low**	-0.088 (0.15)	0.5447	-0.046 (0.011)	<.0001
**APOE ε4-/G-Score high**	-0.128 (0.10)	0.1889	0.0008 (0.007)	0.9129
**APOE ε4-/G-Score low**	ref	ref	ref	ref

Abbreviations: se: standard error; G-score: genetic risk score; ref: reference group.

Model adjusted for age, age^2^, sex, sex*age, education, and education*age.

**Fig 3:**
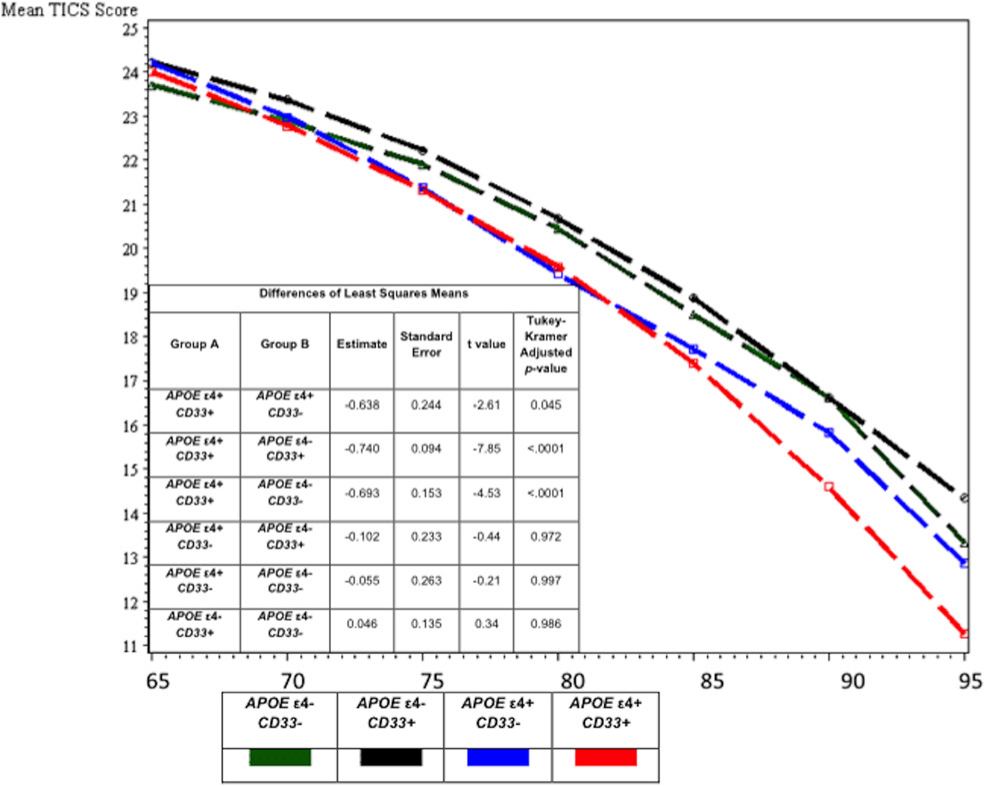
Mixed effects models for cognitive trajectories by *APOE* ε4/*CD33* risk group. Abbreviations: TICS-m: Telephone interview for Cognitive Status-modified. Covariates included age, age^2^, sex, sex*age, education, education*age, group, and group*age.

To further evaluate this phenomenon, we ran post-hoc mixed effects models to evaluate each gene in combination with *APOE* (controlling for age, sex, and education) and found that the increased decline was driven by a statistically significant interaction with *CD33* (-0.68, 95% CI -1.33 to -0.03; p = 0.04). When a Bonferroni correction[17] for multiple comparisons was applied, findings were no longer statistically significant. Separate factorial models were constructed to evaluate *APOE/CD33* combinations in four mutually exclusive groups; *APOE ε4-/CD33-*, *APOE ε4-/CD33+*, *APOE ε4+/CD33-*, *and APOE ε4+/CD33+* (Fig 3). This model was adjusted for baseline age, age*age, sex, sex*age, education, education*age, and interaction terms for the four-level variable*age. The group with both *APOE* ε4+ and *CD33+* was significantly different than the group with only *APOE* ε4+ (-0.638, SE 0.24; Tukey-Kramer adjusted p = 0.045). To rule out effects of the *APOE* ε2 allele, we ran models stratified by *APOE* ε2 status (yes/no) and the association was relatively unchanged in the sample without *APOE* ε2.

## Discussion

In this study of 7,451 participants from the HRS, individuals with one or more *APOE* ε4 alleles and a high g-score appear to experience greater cognitive decline over time than individuals with either *APOE* ε4 or a high g-score alone. Post hoc analyses restricting the sample to individuals in the highest or lowest quartiles of g-score did not produce any meaningful change in the results. However, further investigation revealed that the increased decline in individuals with both *APOE* and other AD risk genes was driven primarily by an interaction between *APOE* and *CD33*. This is the first study we know of to suggest the possibility of an interaction between *APOE* ε4 and *CD33* affecting cognitive function over time in the HRS data, although we acknowledge that the interaction was no longer significant once a correction for multiple comparisons was applied.

Both the *APOE* and *CD33* genes are located on chromosome 19, however these two genes are not in linkage disequilibrium as they are 6Mb apart [3]. The C allele of *CD33* (rs3865444) has been associated with an increased risk of AD in genome-wide association studies [3, 4], has been tied to more severe cognitive decline in AD [18, 19], and correlated with lower MMSE scores [20]. Using data from the Religious Orders Study, Memory and Aging Project, and the Chicago Health and Aging Project, Bradshaw et al found that the C allele accounts for a significant portion of the variance in *CD33* gene expression (>70.0%), is associated with PiB positivity, neuritic plaque burden, and reduced phagocytic activity [21]. The relationship between genotype and gene expression appears to be consistent over the life course, suggesting that *CD33* may be a marker of susceptibility [21]. *CD33* is involved in clearance of Aβ from the brain [22] so one might speculate that the cognitive effects of *CD33* become apparent later in the disease process. This notion aligns with a model of the disease that specifies cognitive deficits as occurring later, after the accumulation of Aβ [23]. Others have speculated that the association between *APOE* and AD decreases with increasing age [24, 25]. These theories both correspond with our model where the effects of *CD33* seem indistinguishable from *APOE* early on but appear to strengthen later. When combined with the cumulative effects of *APOE* later in life, the underlying function of this gene may become more important. Although the actual mechanism is unknown, an interaction between these two genes resulting in potentially increased accumulation of β-amyloid is plausible.

The level of cognitive decline among those with a high genetic risk score in the absence of *APOE* ε4 was not statistically significantly different than the level of decline in individuals with a low genetic risk score. A number of investigators have reported similar results using genetic risk scores calculated in much the same manner[26–28] including those using the HRS cohort [29, 30]. Most of these studies have failed to find significant differences in cognition (including cross sectional performance[26, 27], progression to mild cognitive impairment [26], or dementia outcomes [27, 30]). The current study differs in several important ways. First, the cohort was restricted to ages 65 and older where cognitive decline is more likely to be of a detectable magnitude. Other studies included younger individuals, which may influence the ability to detect cognitive decline [27, 29, 30]. Second, we looked at cognitive decline over time in a cohort with evaluations that took place every two years on average using a global measure of cognition that is heavily weighted to episodic memory. Third, our sample size was larger than most prior studies. And finally, we controlled for education in our models as this is an important covariate in studies of cognitive function at baseline and over time.

### Limitations

The HRS TICS-m has been criticized as a blunt cognitive measure; however for the purposes of this study, it appears to be an adequate measure of global cognitive function over time. In this study, we selected only those who self-reported White race to determine the sample used for genetic analysis, which may result in some misclassification. It is likely that the percentage of Northern European ancestry is relatively high among individuals who self-report as White. In addition, a paper by Reitz et al, suggested that Whites and African Americans share common AD risk genes although the frequencies of some risk alleles may differ [31]. Indeed the frequencies of *CD33* alleles are different in African Americans (0.02) than in Whites (0.34). Given this difference and the fact that the current study is not a GWAS study where the genetic association can be confounded by admixture, it seems appropriate to focus only on White race only. Further, the non-White sample is likely too small to yield significant results. When we tested the full sample including individuals with self-reported African American heritage, results were similar, although we note that the proportion of non-White participants was relatively small. Findings were not significant when run in subsample of non-White participants. This suggests that if there were misclassification, its effects were not meaningful. We note the SNPs selected for this study are based on genes identified in the Alzgene top ten list [8]. While this list is continually updated with information from GWA studies, there are likely genes associated with AD that have yet to be identified. For the current study, we chose to focus on the genes that have the strongest associations in the existing literature.

## Conclusions

The ε4 allele of the *APOE* gene is a potent risk factor for cognitive decline over time. Here we have shown in a sample of White participants from the HRS, that cognitive decline among individuals with one or more *APOE* ε4 alleles in addition to AD risk genes of small effect are greater than the risk associated with *APOE* ε4 alone. This risk appears to be pronounced in individuals who also possess one or more copies of the *CD33* C allele although further study and replication of findings is needed.
