# Correction: Effect of *APOE* and *CD33* on Cognitive Decline

**DOI:** 10.1371/journal.pone.0134887

**Published:** 2015-07-31

**Authors:** Kathleen M. Hayden, Michael W. Lutz, Maragatha Kuchibhatla, Cassandra Germain, Brenda L. Plassman

There is an error in the second sentence of the Description of Variables subsection of the Methods section. The correct sentence is: Genes included: *CLU*, *CR1*, *PICALM*, *MS4A6A/MS4A4E*, *CD33*, *CD2AP*, *ABCA7*, and *BIN1*.

In Fig 2, the table includes incorrect values. Please see the correct Fig 2 here.

**Fig 2:**
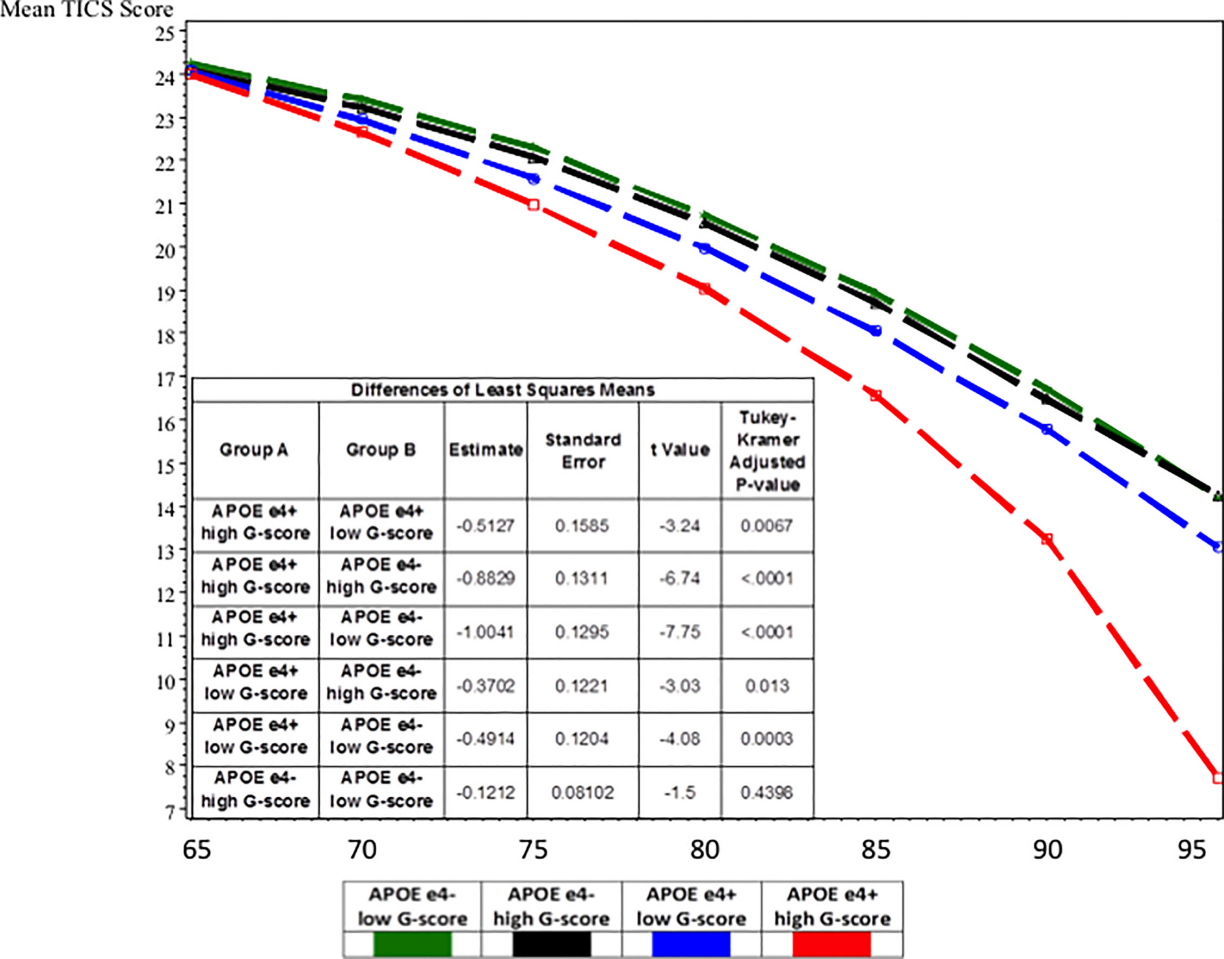
Mixed effects models for cognitive trajectories by APOE ε4/G-score risk group. Abbreviations: TICS-m: Telephone interview for Cognitive Status-modified; high G-score: high genetic risk score; low G-score: low genetic risk score. Model included age, age^2^, sex, sex*age, education, education*age, and group*age. Corresponding model results for the four-level variable and interaction terms are presented in Table 2.
